# Consideration of Metabolite Efflux in Radiolabelled Choline Kinetics

**DOI:** 10.3390/pharmaceutics13081246

**Published:** 2021-08-12

**Authors:** Yunqing Li, Marianna Inglese, Suraiya Dubash, Chris Barnes, Diana Brickute, Marta Costa Braga, Ning Wang, Alice Beckley, Kathrin Heinzmann, Louis Allott, Haonan Lu, Cen Chen, Ruisi Fu, Laurence Carroll, Eric O. Aboagye

**Affiliations:** 1 Cancer Imaging Centre, Department of Surgery and Cancer, Faculty of Medicine, Imperial College London, London SW7 2BX, UK; yl9110@ic.ac.uk (Y.L.); marianna.inglese17@imperial.ac.uk (M.I.); suraiya.dubash@nhs.net (S.D.); chris.barnes@imperial.ac.uk (C.B.); dianabrickute@yahoo.co.uk (D.B.); marta.costabraga@icr.ac.uk (M.C.B.); n.wang16@imperial.ac.uk (N.W.); a.beckley11@gmail.com (A.B.); kathrin_heinzmann@hotmail.com (K.H.); Louis.Allott@hull.ac.uk (L.A.); haonan.lu12@imperial.ac.uk (H.L.); c.chen@imperial.ac.uk (C.C.); ruisi.fu14@imperial.ac.uk (R.F.); lcarro19@jhmi.edu (L.C.); 2 Russell H. Morgan Department of Radiology and Radiological Sciences, Johns Hopkins University School of Medicine, Baltimore, MD 21205, USA

**Keywords:** choline kinase, hypoxia, efflux

## Abstract

Hypoxia is a complex microenvironmental condition known to regulate choline kinase α (CHKA) activity and choline transport through transcription factor hypoxia-inducible factor-1α (HIF-1α) and, therefore, may confound the uptake of choline radiotracer [^18^F]fluoromethyl-[1,2-^2^H_4_]-choline ([^18^F]-D4-FCH). The aim of this study was to investigate how hypoxia affects the choline radiotracer dynamics. Three underlying mechanisms by which hypoxia could potentially alter the uptake of the choline radiotracer, [^18^F]-D4-FCH, were investigated: ^18^F-D4-FCH import, CHKA phosphorylation activity, and the efflux of [^18^F]-D4-FCH and its phosphorylated product [^18^F]-D4-FCHP. The effects of hypoxia on [^18^F]-D4-FCH uptake were studied in CHKA-overexpressing cell lines of prostate cancer, PC-3, and breast cancer MDA-MB-231 cells. The mechanisms of radiotracer efflux were assessed by the cell uptake and immunofluorescence in vitro and examined in vivo (*n* = 24). The mathematical modelling methodology was further developed to verify the efflux hypothesis using [^18^F]-D4-FCH dynamic PET scans from non-small cell lung cancer (NSCLC) patients (*n* = 17). We report a novel finding involving the export of phosphorylated [^18^F]-D4-FCH and [^18^F]-D4-FCHP via HIF-1α-responsive efflux transporters, including ABCB4, when the HIF-1α level is augmented. This is supported by a graphical analysis of human data with a compartmental model (M2T6k + k_5_) that accounts for the efflux. Hypoxia/HIF-1α increases the efflux of phosphorylated radiolabelled choline species, thus supporting the consideration of efflux in the modelling of radiotracer dynamics.

## 1. Introduction

**Figure 1:**
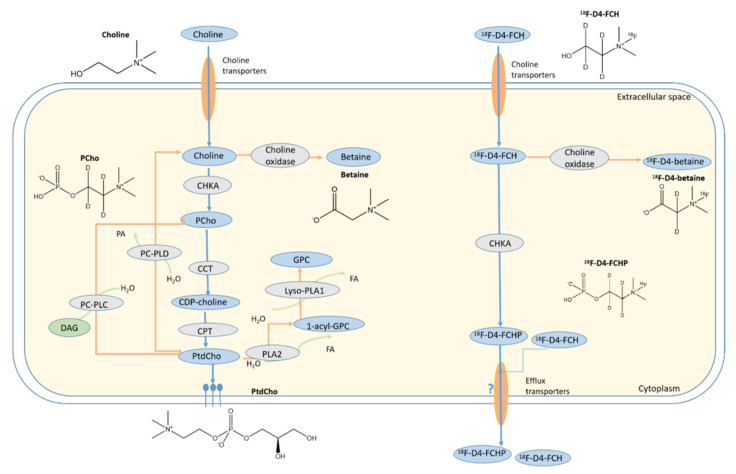
Chemical structures of the choline species and the regulation of choline metabolism. Blue ovals and grey ovals represent the intermediates and enzymes in the pathway. Blue arrows indicate the CDP-choline pathway towards the anabolic synthesis of PtdCho, whereas the arrows in orange indicate the catabolic pathway of PtdCho. Abbreviations: CCT, cytidine choline transferase; CDP-cytidine diphosphate choline; CHPT1, diacylglycerol choline phosphotransferase 1; DAG, diacylglycerol; HIF, hypoxia-inducible factor; FA, fatty acid; GPC, glycerophoshocholine; Lyso-PLA1, lysophospholipase A1; PC-PLC, phosphotidylcholine-specific phospholipase C; PA, phosphatidic acid; PC-PLD, phos-photidylcholine-specific phospholipase D; CTP, choline phosphotransferase; PCho, phosphocholine; PtdCho, phosphotidylcholine.

Choline is essential for the synthesis of membrane phosphatidylcholine (PtdCho) (Figure 1) [1]. It is appreciated that cancer cells, including breast, prostate, and non-small cell lung cancer (NSCLC), alter their choline metabolism in relation to their state of immortalisation and oncogene addiction, with a higher expression of choline kinase α (CHKA) and, concomitantly, high concentrations of phosphocholine (PCho) and the total choline species in malignant metastatic cells compared to normal epithelial cells [2,3,4,5,6].

Consequently, choline metabolism has been exploited as a target for imaging by magnetic resonance spectroscopy (MRS) and positron emission tomography (PET). Several radiotracers have been developed for imaging choline transport and metabolism by PET. These include [^11^C]-choline, approved by the US Food and Drug Administration for imaging recurrent prostate cancer [7,8], as well as a number of fluorinated analogues such as^18^F-fluoromethylcholine ([^18^F]-FCH) developed by the Degrado lab [9,10] and [^18^F]fluoromethyl-[1,2-^2^H_4_]-choline ([^18^F]-D4-FCH) developed by the Aboagye lab [11,12]. Due to a deuterium isotope effect, [^18^F]-D4-FCH has improved the stability to oxidation by choline oxidase reducing the conversion to the corresponding betaine analogue. The stability of deuterated analogues of choline to oxidation by choline oxidase can be explained by the isotope effect embodied in a stronger carbon–deuterium bond strength (in [^2^H_4_]methane) relative to carbon–hydrogen (in methane); the fluorine atom further contributes to the reduced oxidation, as demonstrated by the reduced protection of [^11^C]-D4-choline compared to [^18^F]-D4-FCH [13]. When PET scans with radiolabelled choline tracers, including [^11^C]choline, are conducted dynamically over time, however, almost all tumours exhibit a rapid radiotracer uptake phase followed by a nearly unchanged time versus radioactivity (TAC) curve, regardless of their stability in oxidation [12,13]. This suggests that other mechanisms may influence the tumour radiolabelled choline biodistribution dynamics [14].

Hypoxia and the related acidosis are important tumour microenvironmental factors that influence choline metabolism [15,16]; however, the regulation of choline transport and metabolism, important for radiotracer localisation, are likely complex. In prostate cancer cells, including PC-3 and LNCaP, hypoxia or anoxia decreases the radiolabelled choline uptake [17], while glucose and acetate-based radiotracers show increases or are unchanged under these conditions [18]. The authors attributed this phenomenon in PC-3 cells to the negative transcriptional regulation of CHKA via the HIF-1α activation of hypoxia-responsive element 7 (HRE7) within the CHKA promoter region. In contrast to this study, another study in the same prostate cell line reported the HIF-1α regulated increase of CHKA expression [19]. While studies focus on the relationship between the CHKA expression and radiotracer uptake or unlabelled intracellular phosphocholine concentration, other hypoxia or HIF-1α-associated mechanisms may be at play.

Using [^18^F]-D4-FCH as an exemplar choline radiotracer (from its availability within our lab, representativeness for choline phosphorylation and to avoid a confound of oxidation), the aim of our study was to investigate how hypoxia or HIF-1α regulates the choline radiotracer dynamics and extend the findings to an understanding of the dynamics of radiolabelled choline localisation in mouse xenografts and human NSCLC recently reported by Dubash et al. [14].

## 2. Materials and Methods

### 2.1. Cell Culture and Drugs

Under the standard incubation conditions unless specified, mycoplasma-negative cells were grown in Dulbecco’s modified Eagle’s medium (DMEM) (Thermo Fisher Scientific, Waltham, MA, USA) supplemented with 10% foetal bovine serum (Sigma-Aldrich, St. Louis, MO, USA), 100-U/mL penicillin and streptomycin (Thermo Fisher Scientific, Waltham, MA, USA), and 2-mM glutamine (Invitrogen, Waltham, MA, USA). Cells were cultured at 37 °C in a humidified atmosphere containing 5% CO_2_ and 21% O_2_ (the standard incubation conditions). ICL-CCIC-0019 (CK2) was synthesised in-house (by NW), as previously reported [20,21]. CoCl_2_ and cyclosporin A (cys A) were purchased from Thermo Fisher Scientific, and ko143 and zosuquidar were purchased from Sigma-Aldrich.

### 2.2. Hypoxia and CoCl_2_ Treatment

For the hypoxia studies, the cells were seeded and incubated under the standard conditions for 24 h. The plates were placed within a Scientific Oxoid^TM^ AnaeroGen^TM^ 2.5-L sachet in the sealed Oxoid Anaerogen Jar AG0025A (both from Thermo Fisher Scientific, Waltham, MA, USA). The O_2_ level immediately fell below 1% within 30 min and maintained this until the reopening of the Oxoid Anaerogen Jar. Anaerobic indicator BR0055 (Thermo Scientific, Waltham, MA, USA) was used to monitor the atmospheric oxygen level in the Oxoid Anaerogen Jar. The Oxoid Anaerogen Jar was placed in an incubator at 37 °C. The cells were incubated for 8 or 24 h. For the molecular analysis, the incubation was terminated by placing the plates on ice and washing twice with ice-cold phosphate-buffered saline (PBS) (BR0014G, Sigma-Aldrich, St. Louis, MO, USA). For the CoCl_2_ studies, the cells were incubated with the compound at 200 µM for 24 h under standard incubation conditions.

### 2.3. Transfection

Seeded cells were transfected with 2.5-nM siRNA (117582, Dharmacon, Lafayette, CO, USA) in antibiotic-free media under the standard incubation conditions for 24 h. Four replicates were repeated in three independent experiments (*n* = 12). Transfection reactions were carried out with the Lipofectamine RNAiMAX Transfection reagent according to the manufacturer’s instructions (Thermo Fisher Scientific, Waltham, MA, USA).

### 2.4. pH_in_ Determination

SNARF^®^-4F-5-(and-6)-carboxylic acid (pKa of ~6.4) (S23920, Thermo Fisher Scientific, Waltham, MA, USA) is a fluorescent intracellular pH probe fluorescing at two emission wavelengths (580 nm and 640 nm), as instructed by the manufacturer. The plates were incubated with SNARF^®^-4F in the dark under the standard conditions for 30 min. The cells were cotreated with 200-µM CoCl_2_ when incubated with SNARF-4F and analysed following manufacturer’s instructions. Six replicates were repeated under each condition (*n* = 6).

### 2.5. Immunoblotting

The cells were harvested by the radioimmunoprecipitation assay (RIPA) (R0278, Sigma-Aldrich, St. Louis, MO, USA) containing a 100× Halt^TM^ protease and phosphatase cocktail (78440, Thermo Fisher Scientific, Waltham, MA, USA), as previously described [19]. Briefly, aliquots of 20-μg protein lysates were resolved on 4–15% mini protean TGX Precast protein gels (Bio-Rad, Hercules, CA, USA) at a constant voltage between 200 V and 250 V. The immunoblots were blotted with 1/1000 primary antibodies against CTL1 (AB 198252), CAIX (HPA024153), CAIX (Ab15086), CHT1 (Ab154186), and HIF-1 (610958), ABCB1 (Ab170904), ABCG2 (Ab207732), and ABCB4 (Ab184878), except β-actin (Ab8227) in 1:10,000. The primary antibodies aforementioned were purchased from Abcam, Cambridge, UK. The secondary antibodies against different species were added in 1:5000. The primary antibodies aforementioned were purchased from Abcam (Cambridge, UK). The densitometry values of the protein were obtained using ImageJ (2018, NIH, Bethesda, MD, USA) and normalised to the corresponding levels of β-actin.

### 2.6. Immunofluorescence

The cells were fixed with 4% formaldehyde (Sigma-Aldrich, St. Louis, MO, USA) at room temperature for 15 min, washed with PBS 3 × 5 min at room temperature, and permeabilised with 100% methanol for 5 min on ice, as previously reported [21]. The slides were incubated with the rabbit monoclonal antibody to ABCB4 (ab184878, Abcam, Cambridge, UK,) at 4 μg/mL in 5% BSA-PBS at 4 °C overnight. The next day, the cells were washed 3 × 5 min PBS at room temperature and incubated with the Alexa Fluro 488 goat anti-rabbit IgG secondary antibody at 1 μg/mL (Life Technology, Carlsbad, CA, USA) in the dark at room temperature for 1 h. The cells were washed with PBS 3 × 5 min and incubated with phalloidin Alexa Fluor 594 nm at 1 μg/mL (Life Technology, Carlsbad, CA, USA) for 20 min at room temperature. The samples were counterstained with ProLong Gold Antifade reagent containing DAPI (Invitrogen, Carlsbad, CA, USA, P-36931). The images were acquired on an Olympus BX51 fluorescence microscope. The filter settings were: DAPI (dichroic mirror DM400, excitation at 300 nm, and emission at 420 nm); ABCB4 (dichroic mirror DM500, excitation at 450 nm, and emission at 515 nm); and phalloidin (dichroic mirror DM570, excitation at 510 nm, and emission at 590 nm). The images of DAPI, ABCB4, and phalloidin were acquired at 250 ms, 5 s, and 100 ms, respectively. The images were processed on Image J bundled with Java 1.8.0_172 (2018, NIH, Bethesda, MD, USA). The images were background-subtracted (thresholding) and corrected with the same graphic correction (pixel min 35–max 225) for consistency.

### 2.7. Standard Uptake Assay

The cells were seeded at the density of 5 × 10^5^ per well in the uptake buffer (4.8 mM KCl, 1.2-mM CaCl_2_, 1.2-mM KH_2_PO_4_, 5.6-mM glucose, 25-mM HEPES, and 1-M Tris, pH 6.8) under the standard incubation conditions for 24 h. After the treatments, the media were aspirated, and the cells were washed twice with a warm (37 °C) uptake buffer. The intracellular uptake of [^18^F]-D4-FCH was counted and decay-corrected using the LKB Wallac 1282 Compugamma Laboratory gamma counter (PerkinElmer, Waltham, MA, USA). The protein concentration of the lysates was determined by the BCA assay (Pierce, Waltham, MA, USA). The data are expressed as a percentage of the incubated dose (%ID) per mg of the protein lysate. Three independent sets of 6 repeats (*e* = 18) for each treatment were carried out, shown in Equation (1):

(1) $$radioactivity\;\left(\%ID\;mg\;protein^{-1}\right)=\frac{sample\;count\;\left(cpm\right)\times 100\%}{total\;sample\;protein\;\left(mg\right)*\;count\;of\;standard\;solution\;\left(cpm\right)}$$

In the pHe-dependent assay, the cells were incubated with 0.74-MBq [^18^F]-D4-FCH in a pH-adjusted uptake media (pH between 6.0 and 8.0) without CO_2_ buffering for 1 h. [^18^F]-D4-FCH uptake was tested under different pHe conditions, and after the treatment with CoCl_2_ or hypoxia exposure, in PC-3 (pHe = 6.8) and MDA-MB-231 (pHe = 7.0). Three independent sets of 6 repeats (*n* = 18) for each treatment were carried out. 

### 2.8. Efflux Assay

The cells were pre-treated with 1-µM zosuquidar hydrochloride (SML-1044, Sigma-Aldrich, St. Louis, MO, USA), 3 µM ko143 hydrate (K2144, Sigma-Aldrich, St. Louis, MO, USA), 10-µM cyclosporin A (cys A) (239835, Sigma-Aldrich, St. Louis, MO, USA) in media containing 0.1% DMSO for 24 h to inhibit ABCB1, ABCG2, or ABCB4, respectively, under the standard incubation conditions. The total radioactivity in the efflux media was measured. Three independent sets of 6 repeats (*n* = 18) for each treatment were carried out.

### 2.9. Radio-HPLC

The cells were incubated with 7.4 MBq [^18^F]-D4-FCH under the standard conditions for 1 h. The detailed method was described in reference [11]. The cell homogenates were analysed by radio-HPLC on an Agilent 1100 series HPLC system (Agilent Technologies, Santa Clara, CA, USA) comprising a gamma-RAM Model 3 gamma detector and Laura 3 software (Lablogic, Sheffield, Yorkshire, UK). A Waters µBondapak C18 reverse-phase column (300 × 7.8 mm, Waters, Milford, MA, USA) was used as the stationary phase, together with a mobile phase comprising solvent A (acetonitrile/water/ethanol/acetic acid/1 M ammonium acetate/0.1 M sodium phosphate; 800/127/68/2/3/10) and solvent B (acetonitrile/water/ethanol/acetic acid/1 M ammonium acetate/0.1 M sodium phosphate; 400/400/68/44/88/10). For each sample analysis, Solvent A was run at 100% for 6 min preceding a gradient increment of solvent B from 0% to 100% between 6 min and 16 min; then, Solvent B was run at 100% for 30 s, followed by a linear gradient decrement from 100% to 0% in 1.5 min; then, Solvent A was run at 100% for 1.5 min. Between two sample runs, the column was washed with 100% A at 1 mL/min for 5 min. ^18^F-D4-FCH and ^18^F-D4-FCHP eluted at 14.5 min and 18 min, respectively.

### 2.10. Animal Work

All animal experiments were performed by following the protocols described in references [22,23]. Mice were anaesthetised with isoflurane/oxygen and injected with approximately 1.1-MBq [^18^F]-D4-FCH via a tail vein cannula. Mice remained anaesthetised during a 1-h dynamic PET emission scan in a thermostatically controlled rig on a small animal Genysis4 PET scanner (SOFIE Bioscience, Culver City, CA, USA). The PET scans of mice over 1 h were acquired in the list-mode format for image reconstruction using the maximum-likelihood expectation maximisation method; the decay-corrected imaging data were exported and analysed by Inveon Research Workplace software (Siemens, Munchen, Germany) for visualisation of the tracer uptake. The tumour radioactivity was normalised to that of the whole body to obtain the normalised uptake value (NUV), as previously reported in reference [22]. The area under the NUV curve from 0 to 60 min (AUC) and the ratio of the NUV at 60 min relative to 3.5 min (fractional retention; FRT) were also calculated.

### 2.11. Patients

The study cohort was reported recently [12] and included seventeen patients (mean age ± SD were 64 ± 11 y; age range 39–84 y; weight ± SD, 68.9 ± 15 kg; weight range, 49.7–93.3 kg) with confirmed NSCLC who underwent [^18^F]-D4-FCH PET/CT [14]. The parent plasma input function was generated by normalisation to the plasma-over-blood ratio and correcting for metabolites. In addition to that, to account for the presence of labelled metabolites, the metabolite TAC in the plasma was used as an input for the graphical and compartmental analyses.

### 2.12. Graphical Analysis 

The standard Patlak plot, widely used in parametric imaging for modelling irreversible radiotracer kinetics in dynamic PET, provides the tracer net influx rate constant *K_i_* as the slope of the regression line [24,25,26], as shown in Equation (2):

(2) $$\frac{C_{tissue}\left(t\right)}{C_{p}\left(t\right)}=K_{i}\frac{\int_{0}^{t}C_{p}\left(\tau \right)d\tau }{C_{p}\left(t\right)}+V$$

where *C_p_* is the time-varying plasma concentration of the tracer (parent plasma arterial input function) (kBq/mL), *C_tissue_* is the time-varying tissue concentration of the parent tracer (kBq/mL), and *V* is the volume of distribution of the parent tracer in the tissue.

The modified Patlak plot, which accounts for the presence of a radiolabelled metabolite, provides a *K_i_*′ calculated according to the following equations at a steady state [27,28], Equations (3) and (4):

(3) $$\frac{A}{C_{Tb}\left(t\right)}=K_{i}\prime \frac{\int_{0}^{t}C_{x}\left(\tau \right)d\tau }{C_{Tb}\left(t\right)}+V\prime$$

where:

(4) $$V\prime =V_{ox}\frac{C_{x}}{C_{Tb}}+\sum^{\;}V_{om}\frac{C_{m}}{C_{Tb}}+V_{b}$$

*A*, *K_i_*′, *C_Tb_*, *C_x_*, *C_m_*, *V_ox_*, *V_om_*, and *V_b_* are the total tissue radioactivity (kBq/mL), *K_i_* for [^18^F]-D4-FCH (mL plasma/s/mL tissue), total blood radioactivity (kBq/mL), radioactivity of the parent compound determined by HPLC (kBq/mL), radioactivity of the metabolite (kBq/mL), the steady-state space of the exchangeable region occupied by the parent [^18^F]-D4-FCH, and the steady-state space of the exchangeable region occupied by the metabolite and blood volume, respectively [25]. For comparison, *K_i_*′ was calculated using the parent and total plasma input functions in the standard and modified Patlak models, respectively. The Akaike Information Criteria (AIC) was used to compare the two graphical methods [29].

### 2.13. Compartmental Analysis 

To investigate the best kinetic model, three different kinetic models were tested: the irreversible two-tissue 3*k* model (2T3*k*) and, for the evaluation of the phosphorylation parameter, the standard and modified irreversible two-tissue compartmental models (2T3*k* + *k*_5_ and M2T6*k* + *k*_5_).

## 3. Results

### 3.1. Hypoxia Does Not Influence Expressions of CHKA and Choline Transporter CTL-1 Proteins

**Figure 2:**
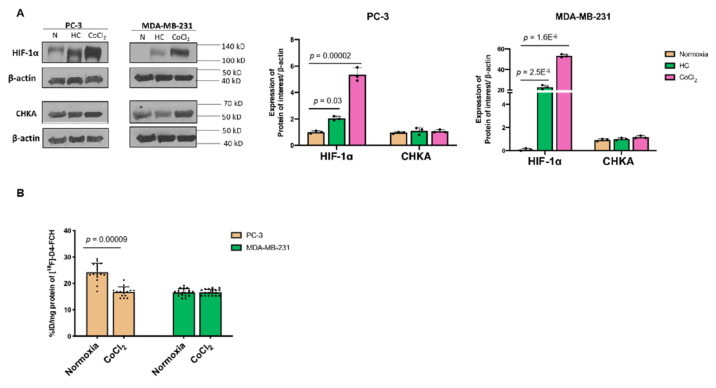
The effects of hypoxia or CoCl_2_ treatment on the CHKA and [^18^F]-D4-FCH uptake. (**A**) Immunoblots of the PC-3 and MDA-MB-231 cells under hypoxia (HC; <1% oxygen for 24 h) and CoCl_2_ (200 µM for 24 h). (**B**) One-hour cell uptake of [^18^F]-D4-FCH after the CoCl_2_ treatment.

We screened a panel of cell lines, including NSCLC (PC-9 and erlotinib-resistant PC-9ER); colorectal carcinoma (HCT-116); and breast cancer (BT474, T47D, and MCF-7) to select the cell lines exhibiting a high ^18^F-D4-FCH uptake (Figure S1A). Two cell lines with the highest ^18^F-D4-FCH uptake, together with detectable CHKA protein expression levels (Figure S1A–C) were selected for investigation of the hypoxia-altered choline metabolic phenotypes: including prostate cancer PC-3 and breast cancer MDA-MB-231. The PC-3 cells were previously used by two laboratories to study the effect of HRE on the *CHKA* expression [17,19]. CoCl_2_ was used to induce HIF-1α by abrogating the hydroxylation function of the prolyl hydroxylase domain (PHD), thus stabilising the HIF-1α protein [17]. Both low oxygen (hypoxia) and CoCl_2_ induced robust increases in the HIF-1α protein (Figure S1A–C); however, only marginal changes of the *CHKA* mRNA levels occurred in PC-3 and MDA-MB-231 cells (data not shown), and their protein expressions were unchanged (Figure 2A and Figure S1B,C).

Studies with CoCl_2_ are subsequently reported, since the protocol allowed HIF-1α induction (without reoxygenation) and [^18^F]-D4-FCH incubation (without a need for a specialised low-oxygen environment). Differences in the metabolic uptake of [^18^F]-D4-FCH were shown between the two cell lines following the treatment with CoCl_2_: there was a 30% reduction of [^18^F]-D4-FCH uptake in the PC-3 cells, while no changes were seen in the MDA-MB-231 cells (Figure 2B). This finding of responsiveness to CoCl_2_ might suggest the existence of different contributions of the CHKA-catalysed conversion of [^18^F]-D4-FCH to [^18^F]-D4-FCHP and/or retention (import and export).

### 3.2. Other Hypoxia-Related Mechanisms to Explain Differences in Uptake

**Figure 3:**
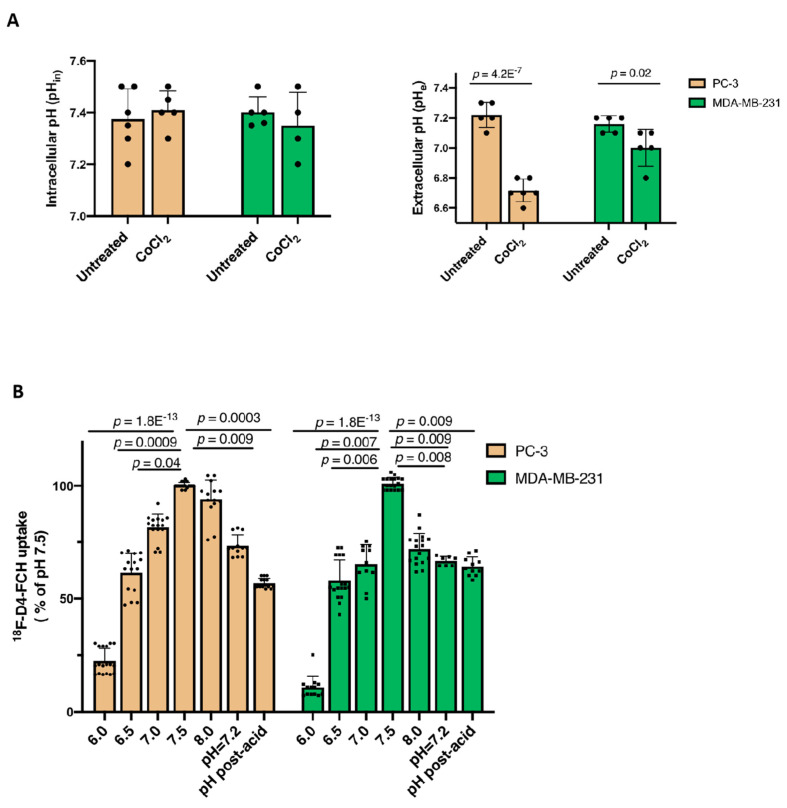
The investigation of the pH on the uptake of [^18^F]-D4-FCH. (**A**) The intracellular pH (pH_in_) and extracellular pH (pH_e_) were assessed in the cells following the treatment with 200 µM CoCl_2_ for 24 h. (**B**) Following a 24 h incubation under normoxic conditions, PC-3 and MDA-MB-231 cells were incubated with 0.37 MBq [^18^F]-D4-FCH in pH-adjusted uptake media (pH_e_ between 6.0 and 8.0) without CO_2_ buffering for 1 h. The data presented are the mean ± standard deviation. One-way ANOVA with post-hoc Tukey’s multiple comparison tests were carried out.

Since the CHKA protein and major transporter CTL1 expressions were unchanged under hypoxia or CoCl_2_, we investigated other potential mechanisms. The upregulation of hypoxia-sensitive marker CAIX (Figure S1B,C) suggested extracellular acidification in hypoxia-exposed PC-3 and MDA-MB-231 cells [28], which led us to examine pH as a mechanism affecting the uptake. The intracellular pH (pH_in_ = 7.3 ± 0.2) in PC-3 and MDA-MB-231 cells remained unchanged post-CoCl_2_ treatment (Figure 3A, left). The induction of extracellular acidification was confirmed (Figure 3A, right). It was shown that the acidified extracellular environment resulted in a 20% reduction of [^18^F]-D4-FCH uptake in PC-3 cells, whilst the uptake was not affected in MDA-MB-231 cells (Figure 3B). The 40% reduction of the cell uptake of [^18^F]-D4-FCH following CoCl_2_ in PC-3 cells was associated with a 38% decrease in the cellular content of [^18^F]-D4-FCHP (Table S1). Radio-HPLC chromatograms are presented in Figure S2. It was possible that the activity of the efflux transporters was influenced by extracellular acidification [30,31]. The substrate spectra of three ubiquitous efflux transporters: ABCB1 (P-GP), ABCG2 (BCRP), and ABCB4 (MDR3), included nearly the entire entity of the antineoplastic agents and other diverse substrates [32,33,34]. Furthermore, we showed that resistance to choline kinase inhibitors can occur via regulation of the ABC transporter expression [33]. Thus, we investigated the contributions of ABCB1, ABCG2, and ABCB4 to the overall efflux of [^18^F]-D4-FCH or [^18^F]-D4-FCHP.

**Figure 4:**
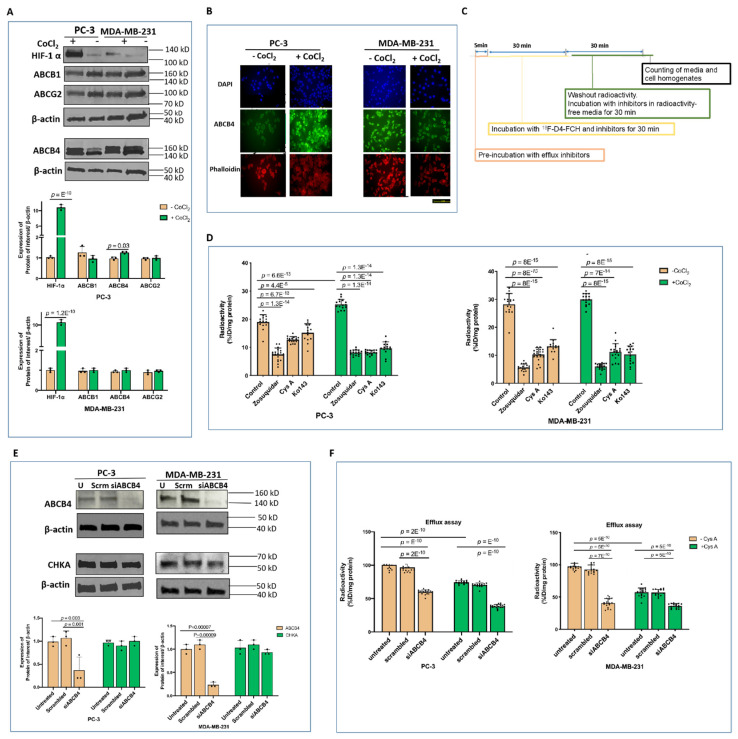
The role of ABCB4 in the modulating efflux of [^18^F]-D4-FCH. (**A**) The immunoblots of PC-3 and MDA-MB-231 cells following the CoCl_2_ treatment. (**B**) Immunofluorescence: DNA stained by DAPI (blue) and cytoskeleton staining by phalloidin (red) and co-stained with ABCB4 (green). (**C**) The workflow of the efflux assay of [^18^F]-D4-FCH. The cells were pretreated with efflux inhibitors ABCB1 (1 µM zosuquidar), ABCG2 (3 µM ko143), or ABCB4 (10 µM cys A) for 1 h in a coincubation with 0.37 MBq [^18^F]-D4-FCH under the standard conditions. (**D**) Efflux assay; the method was described in B. (**E**) Immunoblots of the *ABCB4* knockdowns. Upper, immunoblots of the PC-3 and MDA-MB-231 cells. Lower, densitometry. (**F**) Efflux activity of the radiolabelled choline species of the ABCB4 knockdowns from E.

The effects of the CoCl_2_ treatment on the expression of putative hypoxia-sensitive efflux transporters (ABCB1, ABCG2, and ABCB4) were examined [33,34,35,36], and only the ABCB4 expression in PC-3 cells was altered (Figure 4A). The expression of the ABCB4 doublet band did not change in the MDA-MB-231 cells. In contrast, a large increase of the 160 kD band relative to that of the 140 kD of ABCB4 was seen in CoCl_2_-treated PC-3 cells (Figure 4A). As a result, the overall densitometry of the ABCB4 doublet band in PC-3 cells increased by 30% following the CoCl_2_ treatment. The heavier band of ABCB4 was consistent with the mature form of membrane-bound ABCB4 that completed the post-translational modification and was fully functional as an efflux transporter [37]. The immunofluorescence results suggested that both forms of ABCB4 naturally exist, with the dominant presence of the membrane-bound mature form responsible for export via ABCB4 in the PC-3 and MDA-MB-231 cells (Figure 4B). The CoCl_2_ treatment increased the expression level of membrane-bound ABCB4 in PC-3 cells, in contrast to the expression level of membrane-bound ABCB4 in MDA-MB-231 cells.

**Figure 5:**
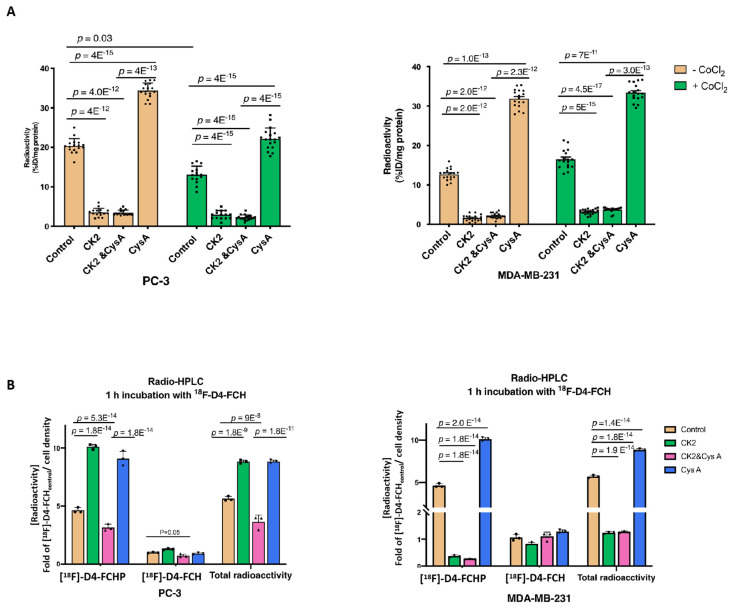
[^18^F]-D4-FCHP featured as a preferable substrate for the efflux system. (**A**) The standard 1-h ^18^F-D4-FCH uptake assay for cells pretreated with CoCl_2_ for 24 h. The Cys A-treated cells were pre-treated with 10 µM cys A for 24 h and then incubated with cys A and 0.37 MBq [^18^F]-D4-FCH for 1 h. Three independent sets of six repeats for each condition (*n* = 18) were carried out. A two-way ANOVA with Tukey’s multiple comparison tests were carried out. (**B**) A summary of the radiolabelled choline species. The dominant radiolabelled choline species [^18^F]-D4-FCH] determines the [^18^F]-D4-FCH uptake. The cells were pretreated with 10 µM CK2, 200 µM CoCl_2_, or 10 µM cys A for 24 h and then incubated with 0.37 MBq [^18^F]-D4-FCH for 1 h. The data are the mean ± standard deviation for three independent repeats (*n* = 3). A one-way ANOVA with Tukey’s multiple comparison tests were carried out.

We designed an efflux assay (Figure 4C) to verify the effect of ABC transporters on the efflux of ^18^F-D4-FCH and to examine the reduction in efflux relative to that of the control cells. In both PC-3 and MDA-MB-231, relatively specific inhibitors for ABCB1 (zosuquidar), ABCG2 (ko143), and ABCB4 (cyclosporin A, cys A) [37,38,39,40,41] decreased the efflux of ^18^F-D4-FCH. The inhibition of [^18^F]-D4-FCH efflux was more pronounced under the CoCl_2_ treatment in PC-3 cells: a 40% difference in the cys A-treated PC-3 cells, while the MDA-MB-231 cells were relatively unresponsive (Figure 4D). ABCB4 is known to efflux the choline-related species PtdCho [42]. The *ABCB4* knockdown profoundly reduced the ABCB4 protein level by over 70% (Figure 4E), leading to a 45% and 70% decrease in the efflux level of the radiolabelled choline species in PC-3 and MDA-MB-231 cells, respectively (Figure 4F). This suggests that ABCB4 is relevant to the ^18^F-D4-FCH efflux in both cell lines; however, in MDA-MB-231, there is no/reduced HIF-1α responsiveness. Cys A inhibited the export of radiolabelled choline species in nontransfected PC-3 and MDA-MB-231 cells and further reduced the efflux level in *ABCB4* knockdown PC-3 cells by 20% (Figure 4F). The potential utility of cys A for rescuing the uptake of [^18^F]-D4-FCH in ABCB4-overexpressing cells was suggested [43,44]. To verify which species was preferentially effluxed [^18^F]-D4-FCH–or [^18^F]-D4-FCHP, we incubated the cells with cys A with or without the CHKA-targeted inhibitor ICL-CCIC-0019 (CK2) [21] to determine the cellular retention of radioactivity independently of CHKA phosphorylation. CK2 reduced the [^18^F]-D4-FCH uptake by >85% in both cell lines (Figure 5A), and under these conditions, the addition of cys A did not significantly influence the total amount of radiolabelled choline species. In the cells without CK2, cys A largely increased the accumulation of the dominant radiolabelled choline species, [^18^F]-D4-FCHP, compared to the relatively unperturbed level of [^18^F-D4-FCH], thereby raising the [^18^F]-D4-FCHP/[^18^F]-D4-FCH ratio to 7:1 in PC-3 and 6:1 in MDA-MB-231 cells (Figure 5B). By inhibiting the efflux of a large proportion of [^18^F]-D4-FCHP, cys A led to a 1.6-fold increase in the total amount of radiolabelled choline species (Figure 5A). Collectively, the findings suggested that [^18^F]-D4-FCHP was the preferable choline substrate for cys A-dependent efflux compared to [^18^F]-D4-FCH. It was further confirmed by radio-HPLC that [^18^F]-D4-FCHP was the predominant choline species for efflux in PC-3 and MDA-MB-231 cells treated with CoCl_2_ (Figure 5B). Our results highlighted a potential utility for cys A to mitigate the HIF-1α-dependent effect on the uptake of [^18^F]-D4-FCH in vitro.

### 3.3. Dynamic ^18^F-D4-FCH PET Data Analysis of NSCLC Tumour

**Figure 6:**
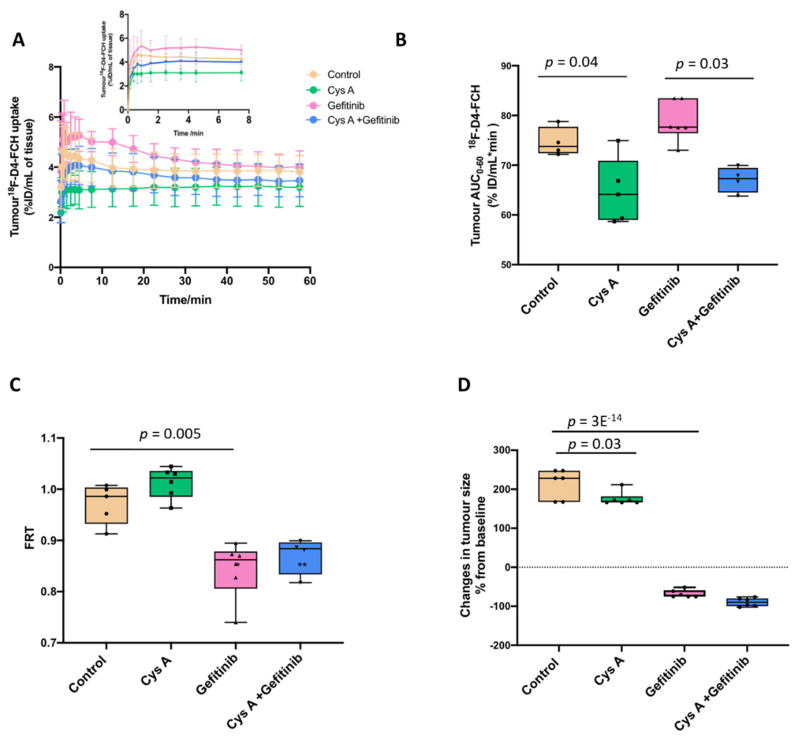
The in vivo 1-h dynamic imaging of [^18^F]-D4-FCH in PC-9 human NSCLC xenograft-bearing mice. (**A**) The time versus radioactivity curves (TACs) in the vehicle control (0.4% Tween 80) or ABC transporter modulator-treated mice; inset: expanded TACs at the early time points. (**B**) Data from the TACs expressed as an area under the radioactivity versus TAC curves, (AUC 0 to 1 h). (**C**) Data from the TACs expressed as the fractional retention (FRT; NUV_60_/NUV_3.5_) across the treatment groups. (**D**) The tumour burden of mice (*n* = 24; *n* = 6 for each treatment) following the 20 mg/kg gefitinib treatment prior to the PET scan. The data presented are the mean ± standard deviation; the average and quartile levels are shown.

We performed [^18^F]-D4-FCH dynamic PET scans in mice to gain an understanding of the tumour kinetics and modulation by ABC transporters. Given that the human dynamic scans for this radiotracer are reported in lung cancer [14], the ABCB4-rich PC-9 human non-small cell lung cancer (NSCLC) xenograft model was used [44]. Mice were treated with a clinically approved drug in this setting, the epidermal growth factor receptor antagonist (EGFR) gefitinib. Of note, EGFR, together with SRC, is mechanistically linked to the activity of CHKA [43]. The effects of the drug and cys A on the tumour uptake of ^18^F-D4-FCH were examined (*n* = 24): the time versus radioactivity curves (TACs) in the vehicle control (0.4% Tween 80) or ABC transporter modulator-treated mice. The radioactivity at the mid-time frames were plotted. All vehicle-treated [^18^F]-D4-FCH PET (control) tumours exhibited a rapid radiotracer uptake phase followed by a nearly unchanged time versus radioactivity curve (Figure 6A). The tumour uptake expressed as the area under the normalised uptake value curve (AUC) was higher with the gefitinib treatment but decreased with the cys A treatment (Figure 6B). We attributed the effects to the broad ATP-binding-cassette (ABC) transporter substrate and enhanced the drug delivery activity of gefitinib [44,45] and the vasoconstrictive nature of cys A in vivo [46,47], in addition to its ABCB4 inhibitory activity. In view of these findings, we also expressed the data as the fractional retention of uptake at 60 min relative to 3.5 min (FRT) (Figure 6C). FRT significantly decreased with the gefitinib treatment, which was in line with the tumour growth inhibition (Figure 6D), with a partial nonsignificant reversal by cys A. FRT may be a useful way to analyse the [^18^F]-D4-FCH PET data to avoid the confounding effects of transporter modulation. Furthermore, a nonvascular-modifying ABCB4 inhibitor may be useful in [^18^F]-D4-FCH PET studies.

### 3.4. Human Lung Cancer Modelling to Verify Efflux Properties of ^18^F-D4-FCHP

**Figure 7:**
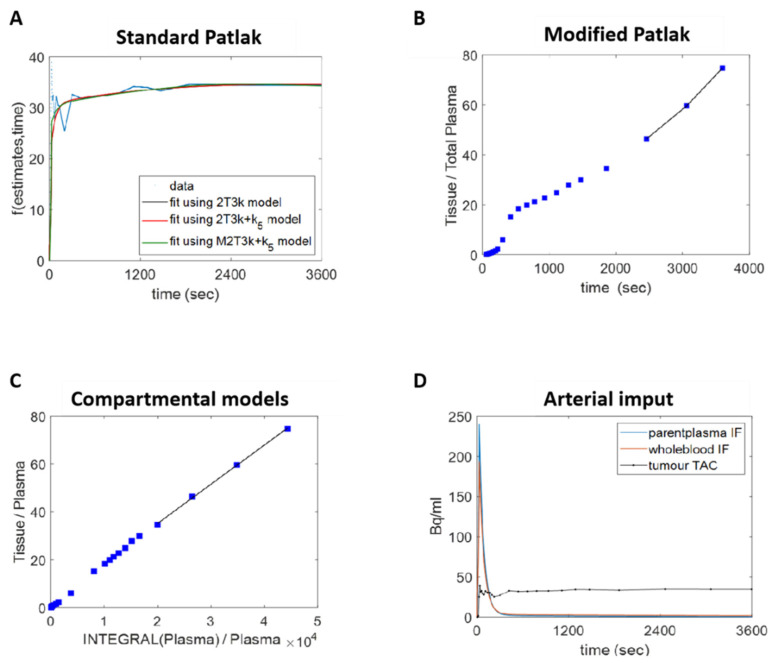
Dynamic PET data quantification. (**A**) The standard Patlak plot assumes the presence of an irreversible compartment and a time t* after which all the reversible compartments are in equilibrium with the plasma. This method provides the irreversible uptake rate constant *K_i_*, which is the slope of the regression line. With the same assumption of the standard one, (**B**) the modified Patlak plot takes into account the contribution of the metabolites to the exchangeable space within tissues and demands the use of an arterial plasma input function that is corrected for metabolites. The *K_i_* is evaluated as before. (**C**) A true quantification of the tracer exchange rates between the compartments can be obtained by fitting the patient data with compartmental models. The irreversible 2 tissue 3*k* (2T3*k*), 2 tissue 3*k* + *k*_5_ (2T3*k* + *k*_5_), and the modified two-tissue 6*k* + *k*_5_ (M2T6*k* + *k*_5_) were used. (**D**) The kinetic analysis of the [^18^F]-D4-FCH PET data demands the implementation of an arterial input function.

The recently reported dynamic PET data from the NSCLC patients (*n* = 17) scanned with ^18^F-D4-FCH were reassessed to investigate the efflux of the [^18^F]-D4-FCH kinetics [14]. The pretherapy ~1 h dynamic [^18^F]-D4-FCH PET/CT scans (maximum dose of 370 MBq) were analysed with graphical and compartmental methods. Of note, all of these therapy naïve tumour lesions showed a rapid uptake and nearly unchanged time versus radioactivity curves (Figure S3A,B). The graphical methods included the standard and modified Patlak plots [24]. The standard Patlak plot assumes a single source for the radiotracer (the parent plasma) and uses a linear model to extract the net influx rate of the irreversible uptake of radiolabelled choline species (Table 1). Distinct from the standard Patlak plot (Figure 7A), the modified model additionally acknowledges the catabolism of [^18^F]-D4-FCH to [^18^F]-D4-betaine and the contribution of the catabolite to the exchangeable space within a tumour (Figure 7B). The compartmental models included an irreversible two-tissue 3*k* model (2T3*k*) (Figure S4A), 2T3*k* + *k*_5_ (2T3*k* + *k*_5_) (Figure S4B), and modified irreversible 2T6*k* + *k*_5_ model (M2T6*k* + *k*_5_) (Table 2 and Figure S4C) [48].

The most parsimonious model fit the data with the fewest number of parameters and entailed a lower AIC value. The AIC values of the graphical methods are reported in Table 1 and testify to the better performance of the modified Patlak compared to the standard Patlak model (*p* < 0.05). The necessity of including the metabolite contribution in fitting the model was also confirmed by the performance of the compartmental models adopted. Table 2 and Table 3 show the comparable AIC values of the 2T3*k* and the 2T3*k* + *k*_5_ models (*p* < 0.05). With the lowest AIC values (Table 4), the most parsimonious compartmental model is M2T6*k* + *k*_5_, which considers both the presence of a radiolabelled metabolite and efflux of the phosphorylation product in the blood/extracellular space. *K*_1_, which describes the delivery of [^18^F]-D4-FCH from the plasma to the extracellular space, is related to tissue perfusion.

The fractional uptake of the parent tracer, quantified with the *Ki*, is an independent parameter and, from the theory, is expected to be comparable in the models adopted [48]. In fact, the two *Ki* values extracted with the standard and modified Patlak show a strong correlation (*R*^2^ = 0.99, Figure 7C). In the comparison between the graphical and compartmental methods, higher *R*^2^ values were obtained when the Patlak *Ki* was compared to the one obtained from the parameters extracted with a model that also took into account the efflux of the phosphorylation product into the blood/extracellular space (Figure 7D). In particular, the strongest correlation involved the 2T3*k* + *k*_5_ model (*R*^2^ = 0.81). The full list of the SUV values evaluated can be found in references [49]. The results of the Spearman correlation test are shown in Figure S5. No statistically significant correlation was found between the parameters extracted with a simplified semiquantitative method and a full kinetic analysis. The Bonferroni method was used for the multiple comparison correction [49]. Thus, while [^18^F]-D4-FCHP efflux appears to be feasible in the patient scans, there is no simple representations of this phenomena in routine clinical static measures.

## 4. Discussion

Radiolabelled choline is approved for imaging cancer and has the potential to be used for monitoring therapy responses. Here, we reported that HIF-1α can reduce the uptake of radiolabelled choline species, exemplified by [^18^F]-D4-FCH, by predominantly effluxing [^18^F]-D4-FCHP through ABC transporters, including ABCB4, and that this finding improves our understanding of the use of radiolabelled choline PET for therapy response assessments. To our knowledge, no studies have reported a relationship between other HIF isoforms and radiolabelled choline uptake.

In the two model systems used, PC-3 and MDA-MB-231 cells, we found variables the effects of HIF-1α modulation on [^18^F]-D4-FCH cellular retention, with PC-3 being the more sensitive cell line. This was despite a similarly robust HIF-1α induction and extracellular pH responses, as well as the expression levels of ABCB1, ABCG2, and ABCB4 in both cell lines. While we appreciate that other ABC transporters may be involved, these three are ubiquitous with wide-ranging substrate spectra [38,39,40,41]. The importance of ABCB4 in the HIF-1α-mediated response was supported by the following findings: (a) using Western blotting and immunofluorescence, only ABCB4 was modulated by HIF-1α, and this occurred only in PC-3 cells consistent with the CoCl_2_-induced reduction in [^18^F]-D4-FCHP cellular accumulation and (b) the use of ABC transporter inhibitors or the genetic knockdown of ABCB4 reduced the efflux of radiolabelled choline species in both cell lines, indicating that a forced modulation of the transporter function can alter the radiolabelled choline uptake. From the ABC transporter inhibitor studies, it appears that the other transporters, including ABCG2 and ABCB1, have the potential to efflux [^18^F]-D4-FCHP, but these were not modulated by HIF-1α. In parallel with the unique function of ABCB4 in mediating phosphatidylcholine (PtdCho) secretion at the canalicular membrane of hepatocytes [50], we found that [^18^F]-D4-FCHP is the more likely of the two species, [^18^F]-D4-FCH and [^18^F]-D4-FCHP, to be effluxed by ABCB4, suggesting the ability of the transporter to efflux not only PtdCho but also the PCho species. The use of radiolabelled choline radiotracers in PET posits the retention of [^18^F]-D4-FCHP within the imaging window of approximately 1 h as the basis for the PET signal; thus, it was surprising that this species could be effluxed under certain physiological contexts or with drug modulators. We speculate that the efflux mechanism partly explains the unique TAC curve of the radiolabelled choline radiotracers in human tumours and, additionally, that HIF-1α accounts at least in part for the spatial heterogeneity of [^18^F]-D4-FCH PET, as previously reported [14]. The PET data from the human tumour study support the efflux mechanism in human tumours [14]. The above findings have implications for the use of [^18^F]-D4-FCH and other radiolabelled choline tracers for studying the therapy response. Cancer drugs that modulate hypoxia or ABC transporter activity can, in principle, also modulate [^18^F]-D4-FCH beyond their direct antitumour activity. The modulation of [^18^F]-D4-FCH uptake by the ABC transporter modulator gefitinib in a NSCLC xenograft supports this fact [45]. We propose a ratiometric approach (FRT) for the analysis of such response data and/or the use of an ABCB4 transporter inhibitor. Of note, the use of ABCB4 transporter inhibitors that do not have vasoactive properties will be preferred for this application.

In summary, we identified the HIF-1α-mediated induction of ABCB4 as a mechanism for effluxing [^18^F]-D4-FCHP species within hypoxic tumours, with implications for the use of [^18^F]-D4-FCH and other radiolabelled choline species for monitoring the anticancer therapy response.

## Figures and Tables

**Table 1:** The results of the standard and modified Patlak plots.

(Mean ± SD)	Standard Patlak		Modified Patlak	
	*K_i_* (×10^−3^)	AIC	*K_i_* (×10^−3^)	AIC
Primary Tumour	1.0 ± 0.4	−231 ± 84	29.1 ± 13.7	−42,259 ± 10,320
Healthy Lung	0.2 ± 0.2	−238 ± 52	7.4 ± 5.8	−48,579 ± 10,297

**Table 2:** The results of the compartmental analysis.

Irreversible Two-Tissue 3*k* Model					
(Mean ± SD)	*K*_1_ (×10^−3^)	*k*_2_ (×10^−3^)	*k*_3_ (×10^−3^)	*K_i_* (×10^−3^)	AIC
Primary tumour	150.2 ± 130.6	54.4 ± 53.0	36.0 ± 19.7	49.7 ± 34.5	−2317 ± 4664
Healthy lung	51.5 ± 59.3	56.7 ± 77.3	55.6 ± 76.5	17.1 ± 16.6	−1845 ± 6508

**Table 3:** The results of the compartmental analysis.

Irreversible Two-Tissue 3*k* + *k*_5_ Model						
(Mean ± SD)	*K*_1_ (×10^−3^)	*k*_2_	*k*_3_	*k*_5_	*K_i_*	AIC
Primary tumour	150.3 ± 130.7	48.8 ± 54.4	156.0 ± 434.5	5.6 ± 7.0	68.5 ± 34.1	−2316 ± 4663
Healthy lung	51.5 ± 59.4	52.9 ± 77.4	65.5 ± 57.7	4.6 ± 5.6	21.2 ± 21.9	−1842 ± 6509

**Table 4:** Result of the compartmental analysis.

Modified Irreversible Two-Tissue 6*k* + *k*_5_ Model								
(Mean ± SD)	*K*_1_ (×10^−3^)	*k*_2_ (×10^−3^)	*k*_3_ (×10^−3^)	*k*_5_ (×10^−3^)	*K_i_* (×10^−3^)	*K*_1__′_ (×10^−3^)	*k*_2__′_ (×10^−3^)	AIC
Primary tumour	153.7 ± 146.9	41.7 ± 62.5	34.5 ± 32.0	13.8 ± 14.5	65.5 ± 49.5	350.3 ± 1042.5	89.1 ± 144.5	2750 ± 5052
Healthy lung	51.0 ± 69.1	1629.4 ± 4797.3	9784.4 ± 26451.3	932.5 ± 2972.4	20.8 ± 24.0	2124.2 ± 3808.5	901.6 ± 1500.3	1369 ± 6236

## Data Availability

All the data presented this study are available from the corresponding author upon reasonable request.

## Supplementary Materials

The following are available online at https://www.mdpi.com/article/10.3390/pharmaceutics13081246/s1: Figure S1. Selection of the cell lines to perform the in vitro assays. Table S1. Summary of the intracellular radio-choline species post-CoCl_2_ treatment. Figure S2. Representative radio-HPLC chromatograms of the PC-3 cells and MDA-MB-231 cells treated or untreated with CoCl_2_. Figure S3: The uptake ratio was evaluated as the ratio between the SUV at 60 and 1.5 min (UR1) and between the SUV at 30 and 1.5 min (UR2). Figure S4: A compartmental analysis. Figure S5: Results of the nonparametric Spearman’s correlation test.
