# Supplementary material for: Consideration of Metabolite Efflux in Radiolabelled Choline Kinetics
Source: Pharmaceutics. 2021 Aug 12;13(8):1246. doi: 10.3390/pharmaceutics13081246 (PMC8400349; doi:10.3390/pharmaceutics13081246)
Supplement: Supplementary file 1 [file pharmaceutics-13-01246-s001.zip › pharmaceutics-1310962-supplementary.pdf]

Supplementary Materials: Consideration of Metabolite Efflux in Radiolabelled Choline Kinetics

Yunqing Li, Marianna Inglese, Suraiya Dubash, Chris Barnes, Diana Brickute, Marta Costa Braga, Ning Wang, Alice Beckley, Kathrin Heinzmann, Louis Allott, Haonan Lu, Cen Chen, Ruisi Fu, Laurence Carroll and Eric O. Aboagye

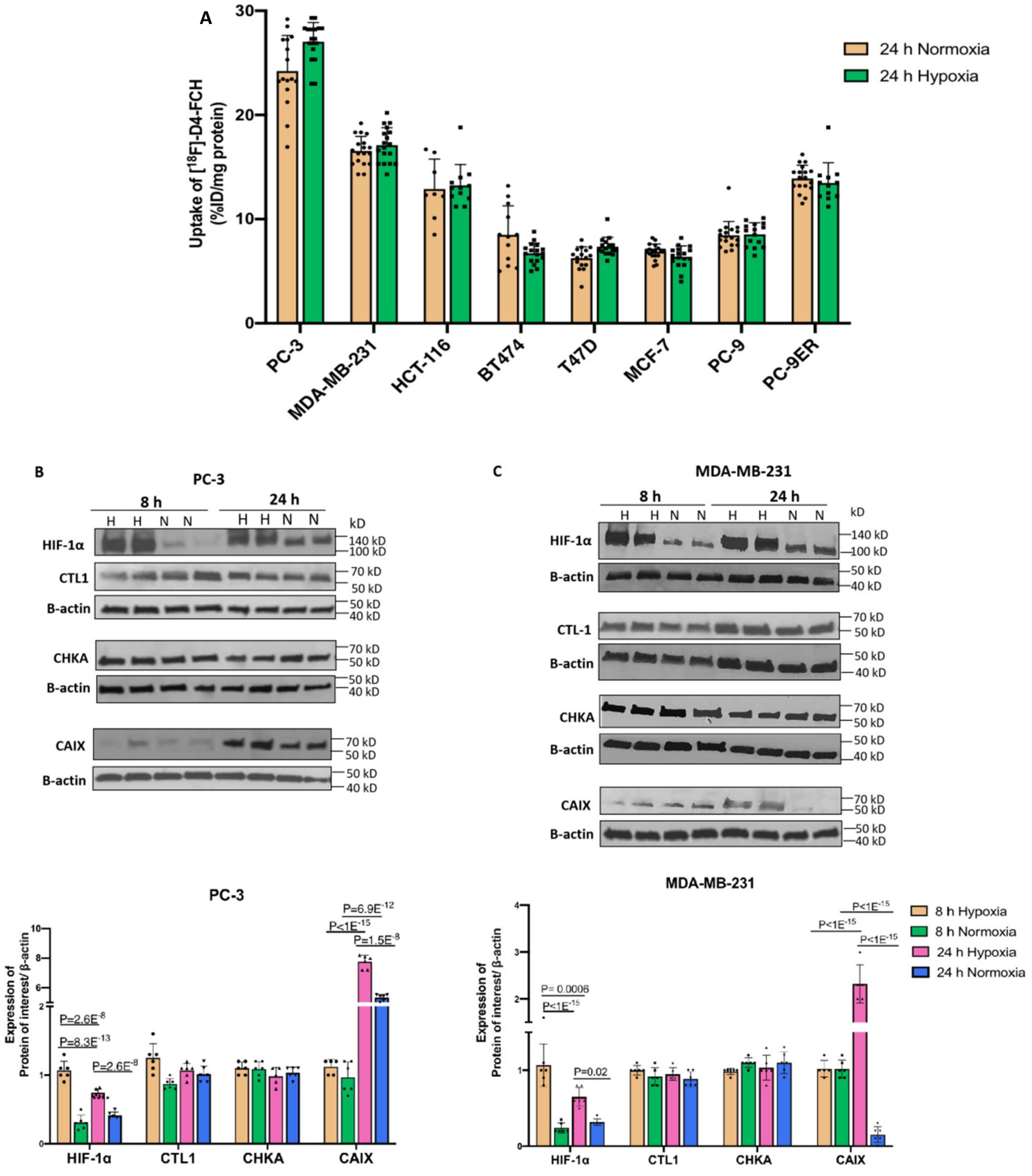

Figure S1. Selection of cell lines to perform in vitro assays. (**A**) Cellular Uptake of [18F]-D4-FCH Across cell lines of breast, NSCLC and Prostate. Regulation of CHKA, CTL1 and CAIX expression under hypoxia. (**B**) PC-3 cells and (**C**) MDA-MB-231 cells exposed to hypoxia within hypoxia chamber. Cells were pre-incubated within hypoxia chamber ($pO_2 < 1\%$) for 8 or 24 h. Under each condition, duplicates were blotted for each protein. One typical duplicate set of three independent repeats was shown in this figure ($n = 6$). One-way ANOVA with Tukey's tests were performed. N and H denoted normoxia and hypoxia, respectively.

Table S1. Summary of intracellular radio-choline species post $CoCl_2$ treatment.

Cell line	Treatment	$A_{[FCHP]}/A_{[FCH]}$	95% Confidential Interval
PC-3	Control	10.01 ± 0.01	9.53, 11.53
	$CoCl_2$ (200 µM for 24 h)	4.02 ± 0.07	3.95, 4.52
MDA-MB-231	Control	3.70 ± 0.41	3.62, 5.67
	$CoCl_2$ (200 µM for 24 h)	2.80 ± 1.08	1.81, 7.17

FCHP:[^{18}F]-D4-FCHP; FCH:[^{18}F]-D4-FCH.

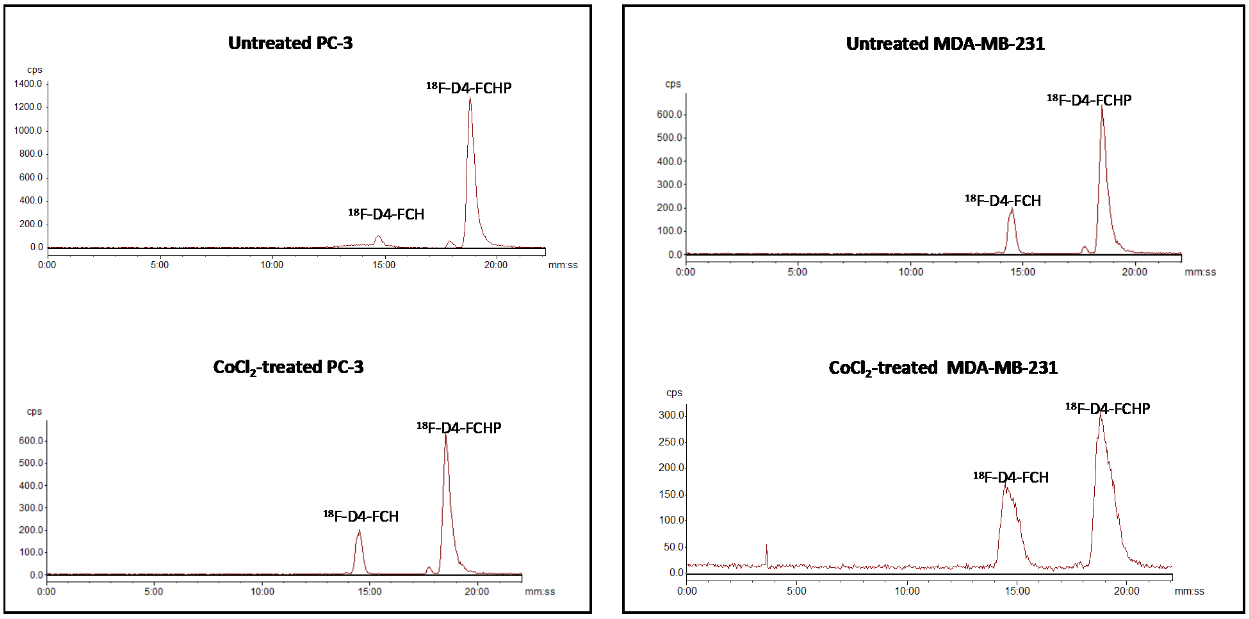

Figure S2. Representative radio-HPLC chromatograms of PC-3 cells and MDA-MB-231 cells treated or untreated with $CoCl_2$.

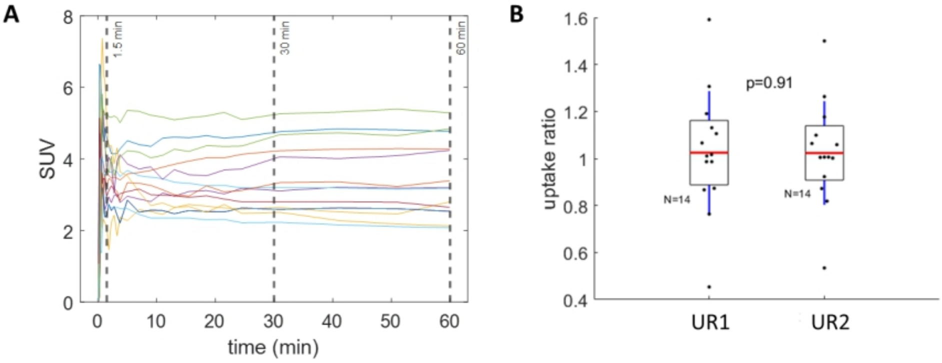

Figure S3. The uptake ratio was evaluated as the ratio between the SUV at 60 and 1.5 minutes (UR1) and between the SUV at 30 and 1.5 minutes (UR2) (**A**) The Wilcoxon rank sum test was used to evaluate the statistical difference between UR1 and UR2 and showed a $p = 0.91$ (**B**).

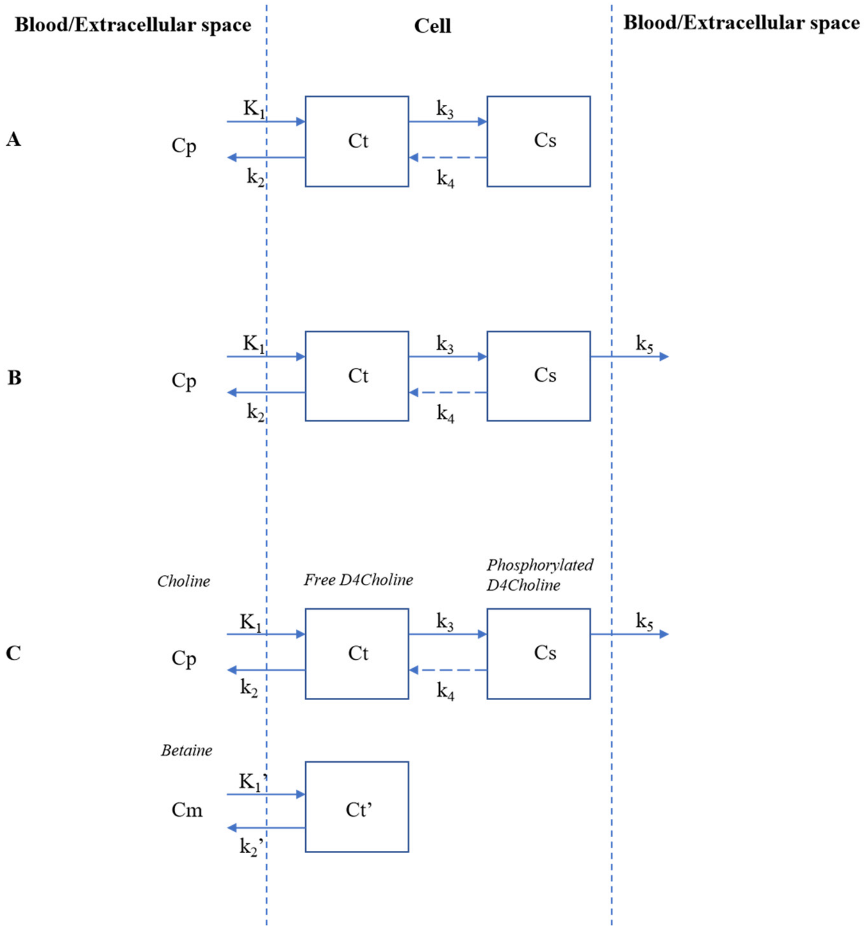

Figure S4. Compartmental analysis. (**A**) The irreversible two tissue 3*k* compartmental model used to describe the exchange of [^{18}F]-D4-FCH into the tissue. (**B**) The irreversible two tissue 3*k*+k_5 compartmental model used to describe the exchange of [^{18}F]-D4-FCH into the tissue and the efflux of the phosphorylation product in the blood/extracellular space. (**C**) The irreversible modified two tissue 6*k*+k_5 compartmental model used to describe the exchange of [^{18}F]-D4-FCH into the tissue, the efflux of the phosphorylation product in the blood/extracellular space and the reversible kinetics of the metabolite (betaine) from the plasma into tissue. C_p is the arterial plasma [^{18}F]-D4-FCH concentration; C_t and C_s represent free and phosphorylated ^{18}F-D4-FCH in the tissue, respectively; C_m is the arterial plasma betaine concentration. K_1 (mL min^{-1}mL^{-1}) and k_2 (min^{-1}) are the parent tracer (and metabolite: K_1', k_2') exchange parameters between plasma and tissue; k_3 (min^{-1}) is the rate at which [^{18}F]-D4-FCH is phosphorylated, k_4 (negligible in this case) is [^{18}F] -D4-FCH dephosphorylation rate constant and k_5 (min^{-1}) is the rate of efflux of the phosphorylation product in the blood/extracellular space.

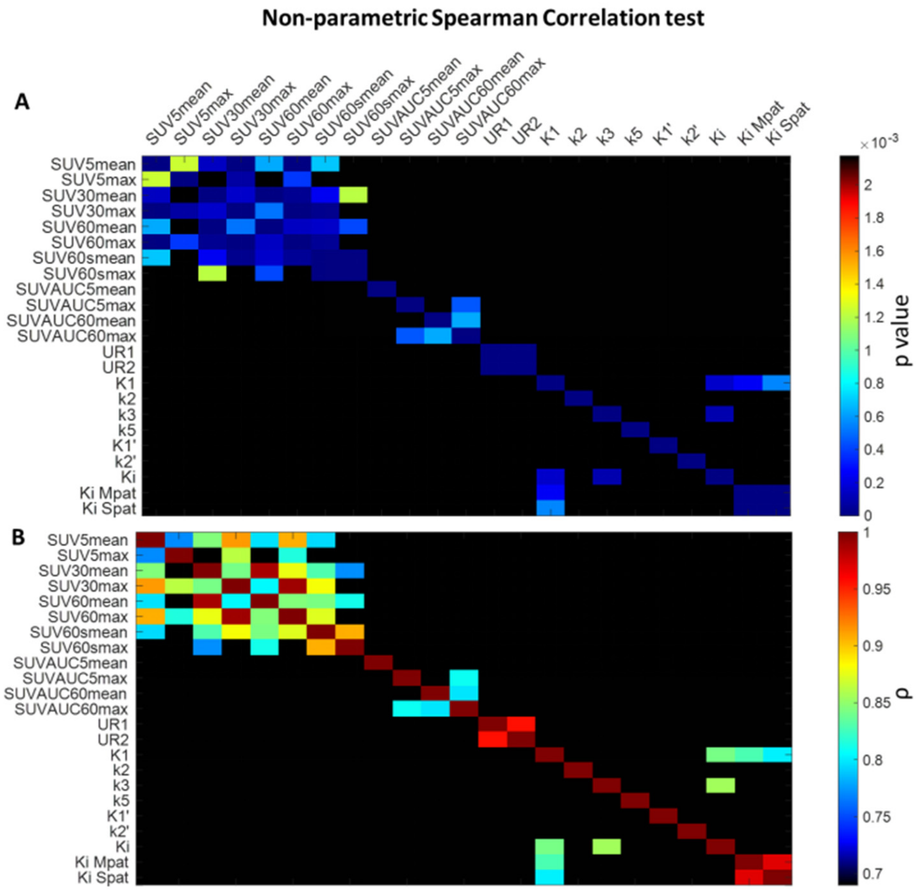

Figure S5. Results of the non-parametric Spearman correlation test. The maximum and mean SUV value at 5, 30 and 60 min from the dynamic acquisition, the maximum and mean SUV value evaluated from the static PET acquisition and the SUV_{AUC} mean and maximum evaluated at 5 and 60 minutes and the uptake ratio evaluated as SUV60min/SUV1.5min (UR1) and SUV60min/SUV1.5min (UR2) were compared to parameters extracted with the graphical standard and modified Patlak and with the M2T6k+k_5 compartmental model (K_1, k_2, k_3, K_1', k_2',k_5 and K_i). Correction for multiple comparison was done with the Bonferroni (statistical $p < (0.05/23)$). The statistically significant p values are shown in (**A**) and the correspondent ϱ values in (**B**).
